# Policy coherence to achieve the SDGs: using integrated simulation models to assess effective policies

**DOI:** 10.1007/s11625-017-0457-x

**Published:** 2017-07-26

**Authors:** David Collste, Matteo Pedercini, Sarah E. Cornell

**Affiliations:** 1 Stockholm Resilience Centre, Stockholm University, Stockholm, Sweden; 2 Millennium Institute, Washington, DC USA

**Keywords:** Sustainable development goals, SDGs, Agenda 2030, System dynamics, policy coherence, Integration, Trade-offs, Synergies, National development planning

## Abstract

Coherently addressing the 17 Sustainable Development Goals requires planning tools that guide policy makers. Given the integrative nature of the SDGs, we believe that integrative modelling techniques are especially useful for this purpose. In this paper, we present and demonstrate the use of the new System Dynamics based iSDG family of models. We use a national model for Tanzania to analyse impacts of substantial investments in photovoltaic capacity. Our focus is on the impacts on three SDGs: SDG 3 on healthy lives and well-being, SDG 4 on education, and SDG 7 on energy. In our simulations, the investments in photovoltaics positively affect life expectancy, years of schooling and access to electricity. More importantly, the progress on these dimensions synergizes and leads to broader system-wide impacts. While this one national example illustrates the anticipated impact of an intervention in one specific area on several SDGs, the iSDG model can be used to support similar analyses for policies related to all the 17 SDGs, both individually and concurrently. We believe that integrated models such as the iSDG model can bring interlinks to the forefront and facilitate a shift to a discussion on development grounded in systems thinking.

## Introduction: the challenge of integration

The Agenda 2030 resolution includes 17 sustainable development goals (SDGs) that are described as *integrated* (United Nations 2015). This implies that the goals, and the effectiveness of the policies addressed to achieve them, depend on each other. Implementation efforts that isolate goals one by one and overlook these systemic interdependencies may hardly be fit for purpose. There are many efforts underway to measure sustainability progress, but to date these have been focused on measures of national and regional asset stocks, or ‘capitals’ (Dasgupta et al. 2015; Managi 2017). Instead, there is a need for integrative approaches that are capable of analysing and elucidating the dynamic effects of interdependencies. This need for approaches grounded in systems thinking has earlier been emphasized in the System Dynamics literature (Barney 2002; Richardson 2005; Kopainsky et al. 2010; and Saeed 2016).

An integrative implementation approach typically begins with identifying causal relationships between goals and policies. Nilsson et al. (2016) propose a simple framework for rating such relationships between SDG targets along a scale of interaction (also in International Council for Science 2016). Their ratings are: −3 cancelling, −2 counteracting, −1 constraining, 0 consistent, +1 enabling, +2 reinforcing, and +3 indivisible. Although useful as a first step in the conceptualization of linkages among the SDGs, the Nilsson et al. framework would benefit from being complemented with more quantitative and integrative simulation tools that support policy analysis. Such tools may complement the framework by enabling connections to be traced across several policies and targets, and identifying probable system-wide impacts of different policy choices.

Designing coherent policies requires acknowledging the corresponding system’s feedback structure. A feedback is a chain of causal relationships that leads back to its origin. For example, if a country invests in education, this may over time, cause a more skilled labour force which may increase productivity. With an effective tax system, this increased productivity could lead to higher government revenues which enable new educational investments. This example of a virtuous cycle of education and productivity improvements involves significant delays, which may need to be considered for successfully assessing the long-term effects of policy choices. From a systems perspective, a multitude of such feedback loops act concurrently to shape a country’s development (Wolstenholme 1983; Richardson 2005; Dangerfield 2008; Qureshi 2009; Kopainsky et al. 2010).

Integrated simulation tools assist policy makers in system-wide policy planning. Such tools or *models* may be considered as bookkeeping units where feedback structures have been identified and translated to conceptual maps and equations that capture dynamic behaviour. Accompanied by scientific insights about various relationships and enriched by data, models can be seen as policy ‘flight simulators’ (Richardson 1997; Sterman 2000; Sterman et al. 2013). Global System Dynamics simulation models have been used to assess problems relating to global commons and limits of material and population growth (Forrester 1971; Meadows et al. 1972; Meadows et al. 2004). Such models may, however, be too blunt and general to be used as tools for assessing the consequences of particular policies on national or sub-national levels, as they do not sufficiently match the policy makers’ geographical scope and level of direct influence.

As most relevant policy making takes place on regional, national and sub-national levels, models that can bridge scales may be particularly useful (Häyhä et al. 2016).

In this paper, our objective is to demonstrate how integrated simulation models may be used to understand and develop scenarios to study synergies and trade-offs for progress on the SDGs on the national level. We present the newly developed *Threshold 21 iSDG model*. iSDG is a flexibly structured System Dynamics based model designed to explore scenarios for policy integration to achieve the SDGs. It builds on the well-vetted Threshold 21 model that has been applied to over 40 nations and has evolved over the past 30 years through research and application (Barney 2002; OECD 2016). The models are developed by the nonpartisan non-profit organization Millennium Institute (2017). iSDG is designed for regional, national and sub-national policy development, and is typically customized to be applicable to the specific contexts where it is to be used.

In this paper, we present an iSDG model with the focus on three SDGs: 3 (on health), 4 (on education) and 7 (on energy). These goals have clear causal interlinkages and relate to both socioeconomic and environmental aspects of sustainable development. Focusing on three goals assists in identifying potential synergies and bottlenecks related to these particular goals. We use one indicator for each goal: life expectancy for SDG 3, average years of schooling for SDG 4 and access to electricity for SDG 7.

To clearly demonstrate the model, we have chosen to zoom in on one country, Tanzania. In broad strokes, Tanzania is a low-income country in Sub-Saharan Africa, ranked 151 out of 182 countries in the UN’s Human Development Index (United Nations Development Programme 2015). According to World Bank data, 43.5% of the population lives on less than $1.25 a day (on a purchasing power parity basis) (World Bank 2015). Electricity access was 15.3% in 2012 and average years of schooling 5.81 years (Barro and Lee 2015; World Bank 2016).

As a policy intervention to study scenarios, we use investments in photovoltaics. Investments in photovoltaics are directly relevant to SDG 7 on energy, are highly relevant to the environmental dimension of sustainable development, and substantial energy investments has been put forward as an enabler for both social and economic development (Modi et al. 2006). Thereby, we expect that impacts on SDG 7 also will affect the progress on SDGs 3 (health) and 4 (education). Furthermore, as a renewable energy source with limited emissions we do not expect clear counteracting effects, such as reduced air quality, which would likely have been the result of coal plant investments. In the subsequent simulations, we identify the expected effects of yearly investments of 1–3% of GDP in photovoltaics, between 2015 and 2031.

## Materials and methods: system dynamics and the iSDG model

### System dynamics

System Dynamics is a discipline and a systems analysis approach that is used to study behavioural patterns of systems. The behavioural patterns are analysed as the outcomes of complex systems in which variables are causally connected in feedback loops. Models are constructed as simplified representations of real-world systems, and are used to facilitate learning about the hypothesized causal structure and behaviour of the real-world systems. System dynamics typically use both cognitive maps, such as causal loop diagrams, and simulation models. In the simulation models, the mathematical representations are combined with interfaces that make the assumptions about causalities explicit. This typically enables exploring different what-if questions and performing sensitivity tests to explore potential system-level leverage points. In mathematical terms, system dynamics models consist of series of integrals, also referred to as stocks or accumulations; and derivatives, referred to as flows (Axelrod 2003; Ford 2009; Sterman 2000; Meadows 2008; Richardson 2005).

Variables that are related to the iSDG model can be separated into three categories:

- *Endogenous variables* are variables that are derived from (that is, depend on) other variables from within the model.
- *Exogenous variables* are given from outside the model, and
- *Excluded variables* are variables that are not included in the model.

(Following the approach of Sterman 2000; Ford 2009).

Building confidence in a system dynamics model entails ensuring its causal relationships are credible. Qualitative aspects of the model are, therefore, often in focus. Model validity requires having a thorough and well-supported theory of causality in addition to more quantifiable validation criteria (Barlas 1996).

### The iSDG model

**Fig. 1:**
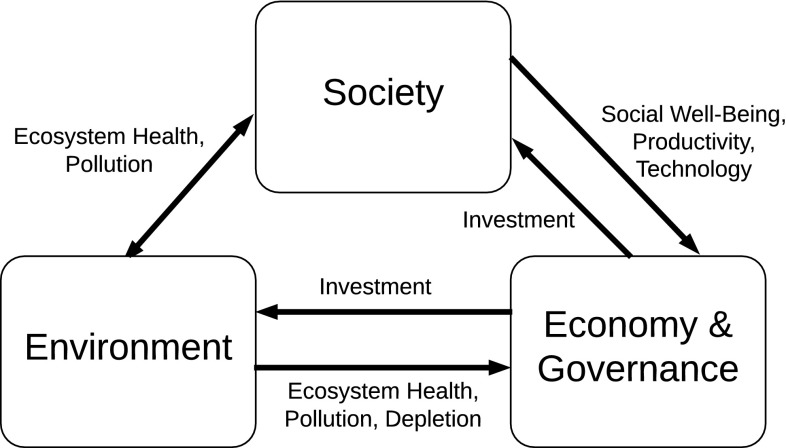
Main sectors of the iSDG model. Based on Barney (2002)

The iSDG model is designed to assist in development planning by providing a credible representation of real-world development. iSDG, like its forerunner Threshold 21, is based on feedbacks between and within three main sectors that may be referred to as *environment*, *society* and *economy and governance*, Fig. 1.

**Fig. 2:**
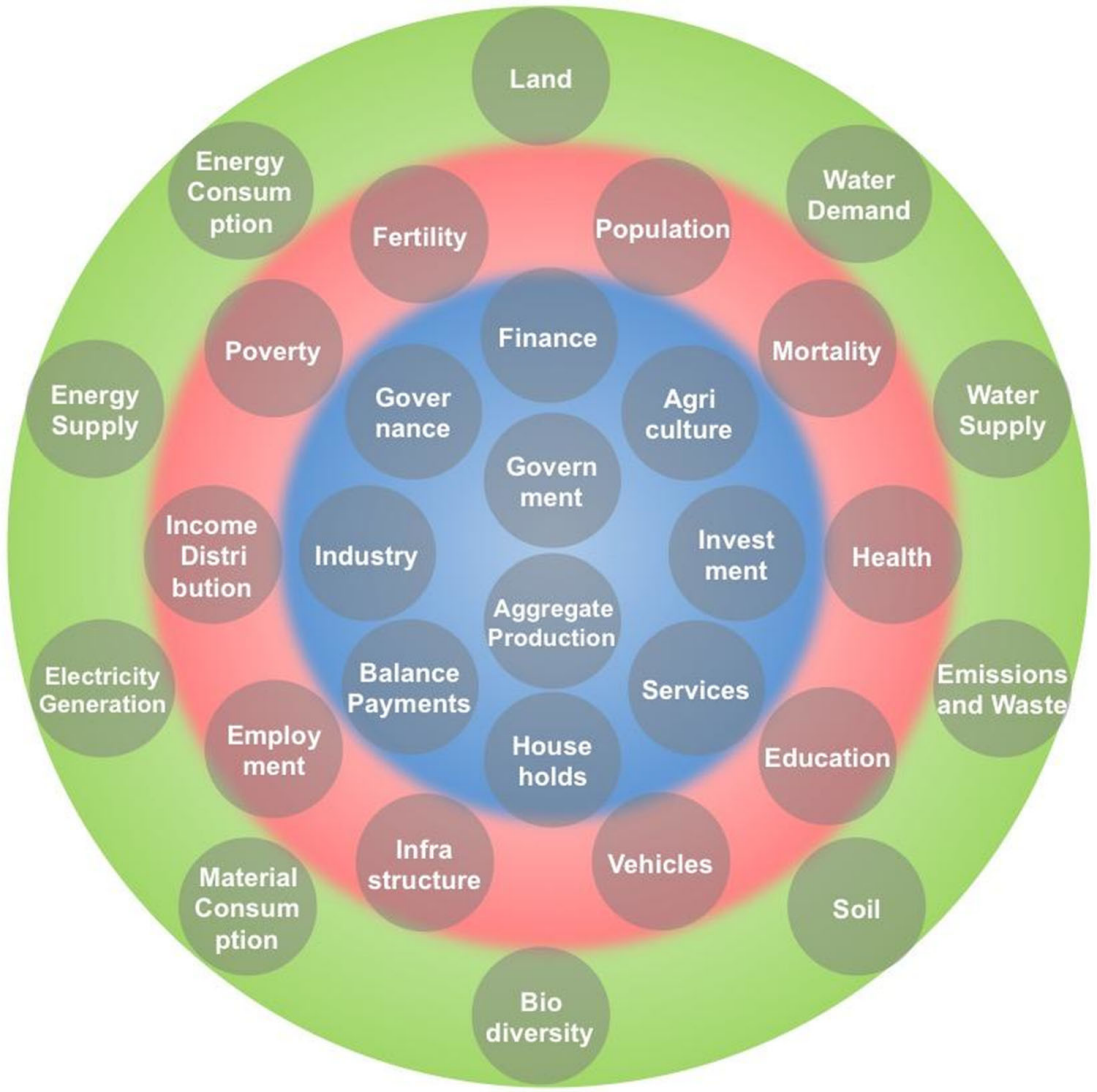
Overview of the iSDG subsectors. The *outer green* field includes the environment subsectors, the *middle red* field the society subsectors and the *inner green* field the economy and governance subsectors. Source: Millennium Institute (2016)

Each sector consists of 10 subsectors, as displayed in Fig. 2. Within these sectors, the iSDG model includes more than 1000 stock variables. It is, therefore, not possible here to give a detailed presentation of the entire model. Instead, we outline a few simplified examples of model structure when explaining the key components of our demonstration case of the effects of investments in photovoltaics and the feedbacks between SDGs 3, 4 and 7 for Tanzania. Documentation of the iSDG model structure can be found at http://isdgs.org (Millennium Institute 2016), and the full model may be shared upon request.

Variables in focus for development planning are modelled as endogenous. These include for example aggregate production, population, the demand and supply of energy, and their determinants. Modelling these variables as endogenous enables the model to be used to explore a systems perspective of development. The allocation of public resources between different subsectors of government is typically modelled exogenously, to enable the exploration of alternative scenarios for national development planning, by varying the budget allocations.

The adoption of Agenda 2030 and the increased availability of relevant literature and data have supported enriching the iSDG model structure with additional relationships between various SDGs. Strengthening the feedback network across the SDGs makes the model correspond better to reality, and provides a more accurate representation of development processes and their contribution to the system’s behaviour. In addition, a better mapping of the relationships between the goals is becoming increasingly relevant both in the academic (Nilsson et al. 2016) and political arenas (United Nations 2015). Strengthening the feedback network may, therefore, also make the model more policy relevant.

In our development of an applied iSDG model, we note prime characteristics of system dynamics model formulations (Forrester 1992; Barlas 1996): the use of diverse data sources and the focus on anticipated causal structure and qualitative aspects of models in model validation.

As the intention is to provide a credible, well-grounded and useful hypothesis of the overall causal structure of a country’s development, *data sources* used are not restricted to numerical data, for example, from national account databases, but can also incorporate other sources of information. These include qualitative theories of causal relationships from literature, and data from diverse experiences provided through expert or stakeholder interviews (Forrester 1992). As the first national customization of the iSDG model, the calibration process of applying the model to Tanzania was based on earlier Threshold 21 models (Kopainsky et al. 2015; UNEP 2015; Allen et al. 2016), relationships included in published papers, and publicly available data. The main numerical data sources used were the World Bank and International Energy Agency. Typically, however, the Millennium Institute’s calibration process also includes interviewing stakeholders, iterating between different possible model formulations, and investigating their respective consequences for the anticipated model behaviour.

Both the quantitative behaviour of the model (its outputs) and its causal hypotheses need to be supported with evidence. With the aspiration to create credible causal hypotheses of national development, *model validation* of the iSDG model includes both comparing the model’s behaviour with data on historical behaviour, and qualitatively and quantitatively studying model formulations in isolation and combined with the rest of the model.

### An example of iSDG model structure: the construction of photovoltaic capacity

**Fig. 3:**
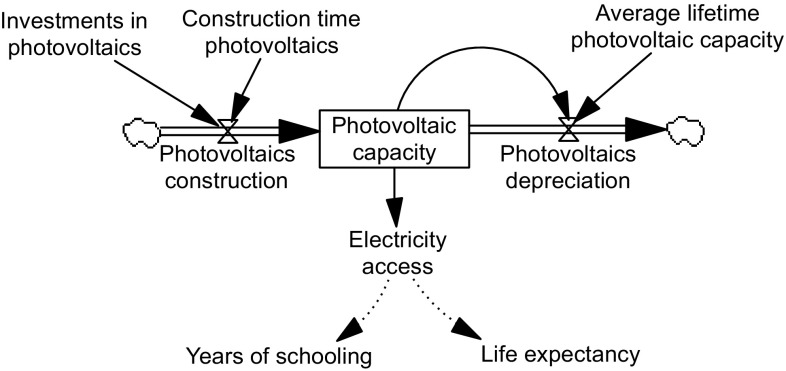
A simplified system dynamics representation of the photovoltaic electricity capacity part of the model

One piece of the new iSDG model structure relates to the construction of photovoltaic electricity capacity, of which a simplified representation is portrayed in Fig. 3.

The arrows in Fig. 3 represent causal relationships. All variables are presented as labels in the Figure. *Photovoltaics construction* is portrayed as a function of the variables *Investments in photovoltaics* and *Construction time photovoltaics*. The more *Investments in photovoltaics*, the more capacity is constructed. The longer the *Construction time photovoltaics*, the slower the construction process. The box in the middle represents *Photovoltaic capacity* as a stock variable, which accumulates over time. The constructed photovoltaics have an average lifetime before they depreciate, represented by the outflow to the right of *Photovoltaics capacity*. Parameter values for all these variables were derived from the International Energy Agency’s estimates. The *Photovoltaics depreciation* flow is a function of *Photovoltaic capacity* and the *Average lifetime photovoltaic capacity.* 1 *Photovoltaic capacity* affects *Electricity access,* which in turn affects *Years of schooling* and *Life expectancy*. These links are discussed below.

The output obtained from running the entire iSDG model was compared with historical data for 1990–2015 for selected variables, including GDP, life expectancy, electricity access and years of schooling. The model output matched the historical behaviour well, which increased our confidence in using the model to explore plausible future scenarios incorporating policies that include significant investments in photovoltaics.

## Mapping causalities

In this section, we explore causal relationships between SDGs 3, 4 and 7, identify model modifications to enable an investigation of an energy system intervention (investment in photovoltaics), and outline how these are incorporated into the iSDG model structure.

### Relationships between SDGs 3, 4 and 7

**Fig. 4:**
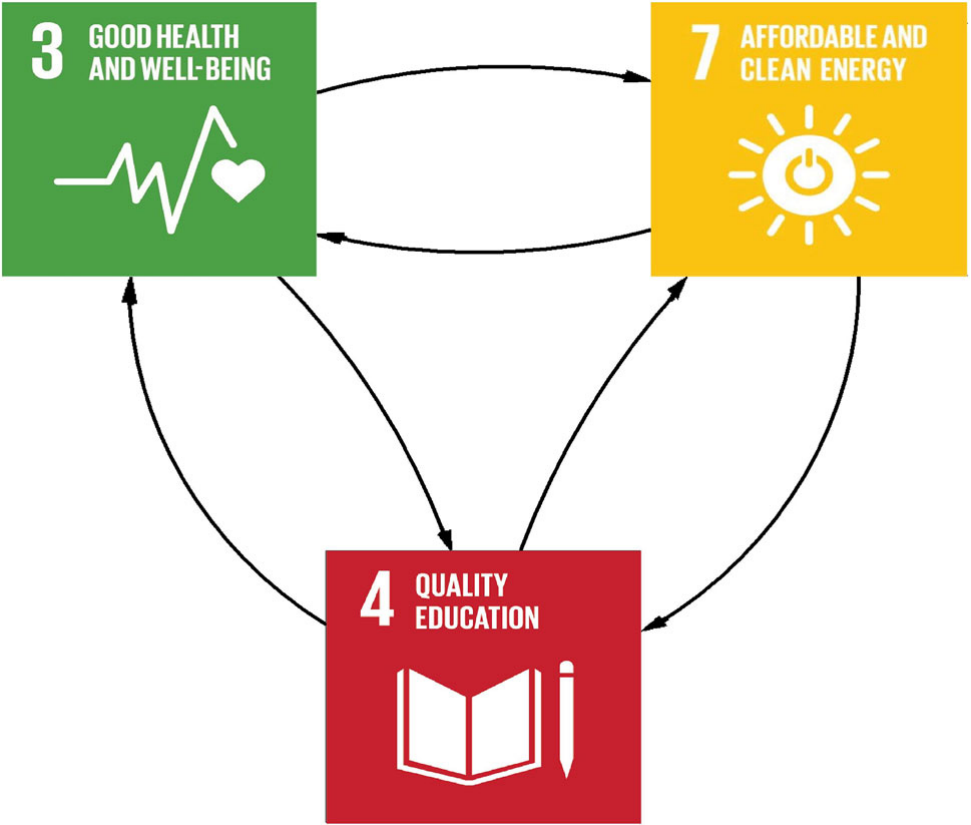
Potential causal chains between electricity access, life expectancy and years of schooling. Note that the many intermediates through which the effects are channelled are not included in the Figure

Causal links between access to electricity, life expectancy and years of schooling may be presented as causal pathways including chains of causal connections where the final outcomes depend on interaction between various factors. By focusing on three of the SDGs we have six such potential causal chains (Fig. 4). The causal chain from education to health has already been included in the earlier version of the Threshold 21 model, so it will not be further discussed here.

Each causal chain may be either positive or negative. For example, *life expectancy* may affect *years of schooling* either positively (that is, *higher life expectancy* causes an increase in *years of schooling*) or negatively (i.e. *higher life expectancy* causes a decrease in *years of schooling*), and *years of schooling* may, in turn, affect *life expectancy* either positively or negatively. Moreover, significant delays between the parts of the chains may exist, e.g. it may take time for improvements in early childhood nutritional status to affect educational outcomes. Below, we go through each causal chain separately.

#### The effect of electricity access on life expectancy

Incorporating a positive causal relationship from electricity access to life expectancy may be justified based on the following reasoning (Abdelkarim et al. 2014; Ezzati et al. 2004; Khandker et al. 2013; Lim et al. 2012; Modi et al. 2006; The World Bank 2008):

- Access to electricity reduces the use of solid fuels and kerosene for cooking and lighting. The use of solid fuels and kerosene for cooking is common practice in many countries. The consequential indoor air pollution causes many diseases and has severe health effects. Electricity access enables the use of alternative sources for heating and lighting, such as electric kettles and light bulbs, and also enables the use of ventilation appliances. There are also health risks related to fuel collection that can be decreased through the provision of electricity.
- Electric appliances may improve food preservation, which both reduces contamination and enables an increase in the variety of foods that are being consumed. Electricity may also enable the use of electric water pumps and water purification techniques. All this is beneficial for health.
- Electricity access enables refrigeration for medical purposes and improves health care infrastructure. For example, refrigerated medicines and vaccines may be stored for longer; health care facilities with electric lighting can be open after dark, and electricity enables the use of many health services and interventions such as x-rays and ultra-sounds.
- With electricity access, information technology can be used to spread public awareness and knowledge related to for example diseases and health practices.

**Fig. 5:**
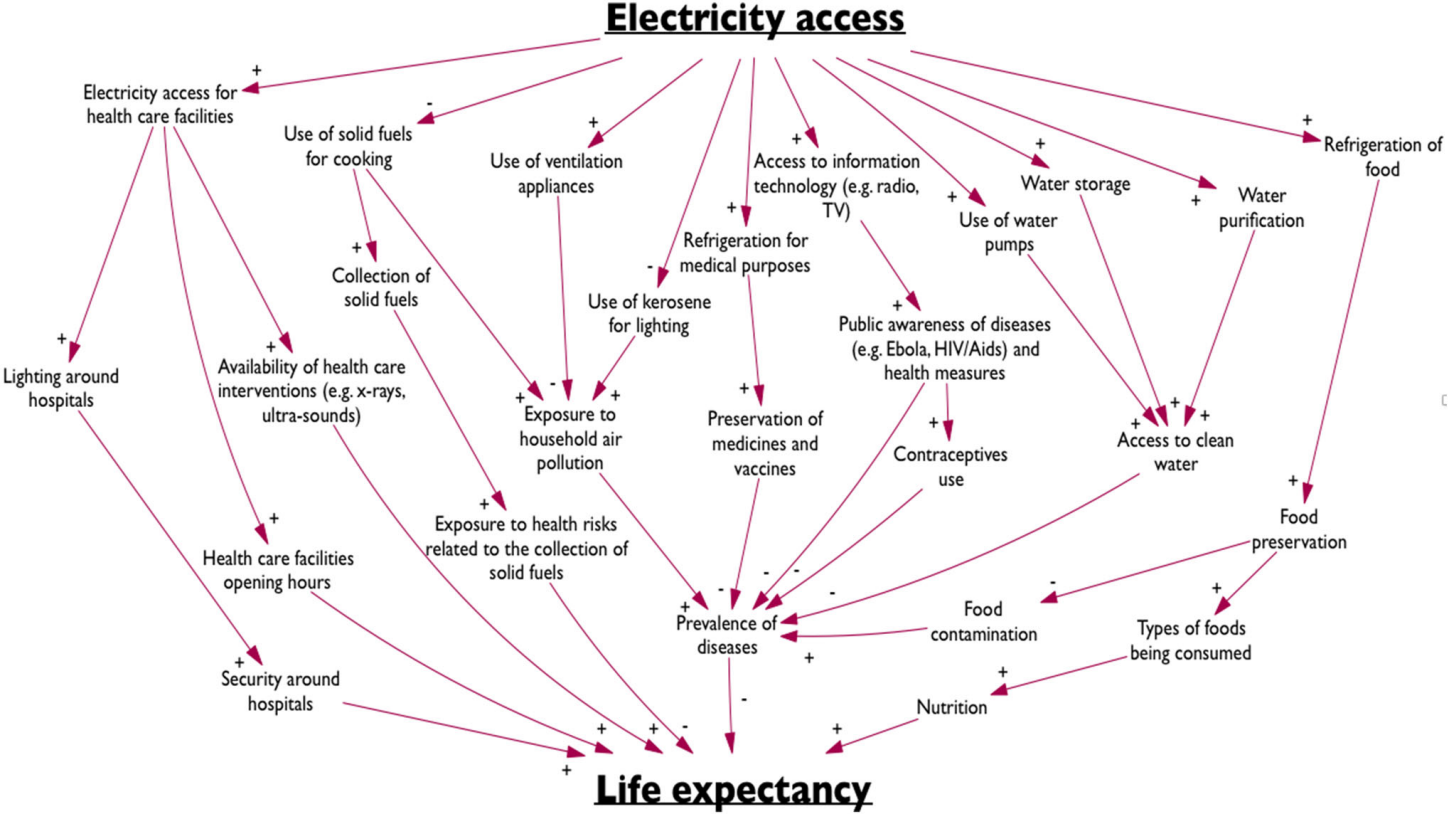
A causal map displaying the relationships between electricity access and life expectancy (referred to as a ‘diamond diagram’). A ‘+’-sign represents a ceteris paribus positive causal relationships (an increase in A causes B to increase, all things equal) and a ‘-‘-sign represents a ceteris paribus negative causal relationship (an increase in A causes B to decrease, all things equal)

The causal pathways between electricity access and life expectancy are summarized in the diamond diagram in Fig. 5. Together, these points clearly indicate a positive causal relationship between electricity access and life expectancy. This is incorporated into the national level application of the iSDG model by a single positive link from *electricity access* to *life expectancy*.

#### The effect of electricity access on average years of schooling

Several arguments point to causal relationships from electricity access to average years of schooling:

- Electricity access enables students to spend more time studying through better light quality, longer duration of lighting, and decreased time spent on collecting water and fuel. A study in Vietnam indicated that electricity access attributed to an increase school attendance by 0.13 years for boys and almost one year for girls (Khandker et al. 2013).
- Learning conditions are improved by access to information communication technologies. Access to electricity in rural areas may also increase the areas’ attractiveness for good quality teachers.

Although the major effects of electrification on years of schooling appear to be positive, the literature also suggests potential negative effects. Abdelkarim et al. (2014) and Modi et al. (2006) suggest that entertainment activities enabled by electricity, such as TV watching, may outcompete studying. Electricity access may also increase the job opportunities in the productive sector, which could affect educational attainment negatively. Provided that these potential negative effects do not dominate, the points together indicate a positive causal relationship from electricity access to average years of schooling. This was incorporated into the iSDG model structure.

#### The effect of life expectancy on years of schooling

The existence of a positive causal relationship from life expectancy (or more specifically, life expectancy as an indicator for health) to average years of schooling may be justified based on the following:

- Healthy students are more present in school, are physically better prepared for studying, and are likely to stay in school for more years. In a study using household survey data from rural areas in China, Zhao and Glewwe (2010) found evidence indicating that children’s nutritional status early in life had a significant effect on completed years of schooling. (Cutler and Lleras-Muney 2006)

This supports adding a causal link from life expectancy to years of schooling in the iSDG model.

#### The effect of life expectancy on electricity access and the effect of years of schooling on electricity access

There seem to be less evidence of causal chains from life expectancy to electricity access, and from years of schooling to electricity access, beside via the productivity effects of health and education. However, we can surmise that a healthy and educated population may take better care of, and upgrade, electrical infrastructure and equipment. Also, education may enable the use of more advanced electrical equipment, which could increase the demand for electricity access.

#### Resulting links

The literature has provided a basis for positive causal chains between electricity access and years of schooling and electricity access and life expectancy. Furthermore, there seem to be bidirectional causality between years of schooling and life expectancy. We did not find strong support for causal chains that go from life expectancy and years of schooling to electricity access, except for via productivity.

**Fig. 6:**
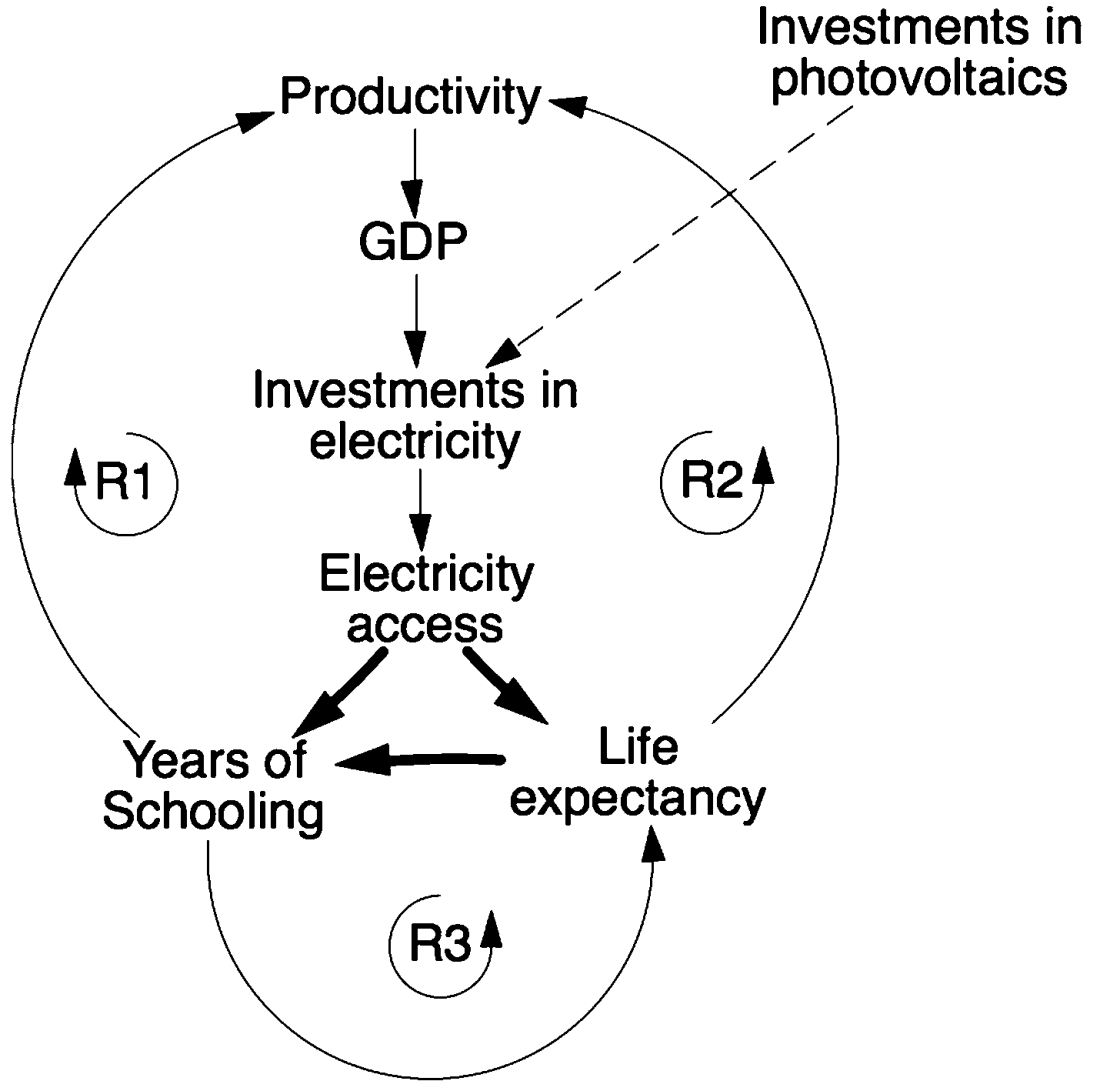
A simplified causal loop diagram displaying the discussed relationships. Each *arrow* represents a positive causal relationship. The *three bold arrows* represent the links that were added to the model. R1, R2 and R3 represents reinforcing loops initiated by the added links

When incorporating the new links into the existing model structure, new reinforcing feedback loops are initiated, displayed in Fig. 6. These three reinforcing feedback loops are labelled R1, R2 and R3. R1 may be referred to as the *Electricity access*—*years of schooling reinforcing loop* and displays that an increase in electricity access causes an increase in years of schooling which, in turn, leads to productivity improvements. Increases in productivity means an increase in GDP which, through increased both government and private funding, enable further investments in electricity which improves its access. R2 could be labelled the *Electricity access*—*life expectancy* reinforcing loop. It displays the assumptions that electricity access improves life expectancy which increases productivity. As is assumed in the R1 loop, improved productivity, over time, causes an increase in electricity access. Finally, the R3 loop that we may label the *Years of schooling*-*life expectancy* reinforcing loop portrays the assumption that improvements in years of schooling are beneficial for health and causes improved life expectancy which, in turn, causes an increase in school attendance. There are significant delays inherent in the feedbacks, e.g. it takes many years for improvements in education to affect a country’s productivity. These delays have been incorporated into the model structure.

The added links make the iSDG model incorporate synergizing impacts between the SDGs 3, 4 and 7. Based on the existing literature we have identified reasonable ranges for parameter values related to these links. We have further indirectly calibrated the relationships by fitting them to historical data for the period 1990–2015 using partial model testing (Homer 2012).

### Incorporating an intervention: investments in photovoltaic capacity

In addition to the causal links added between SDGs 3, 4 and 7, model structure associated with the construction of photovoltaic capacity was incorporated into iSDG. This enables simulating plausible future scenarios that include investments in photovoltaic capacity. Investments in photovoltaics are represented by the dashed line in Fig. 6.

Five different investment policies were considered, ranging from no investments to yearly investments of 3% of GDP (Table 1). All investments are modelled as additional government expenditure, financed through additional financing from financial markets. Accordingly, the policies also imply increased costs for government loans which are endogenous in the iSDG model formulation.

**Table 1:** Policy options explored using the iSDG Tanzania model

Name	Explanation
Policy 1	No expenditure for large scale photovoltaic capacity
Policy 2	1% of GDP expenditures for large scale photovoltaics 2016–2031
Policy 3	3% of GDP expenditures for large scale photovoltaics 2016–2031
Policy 4	0% of GDP expenditures for large scale photovoltaics 2016–2020 1% of GDP expenditures for large scale photovoltaics 2020–2025 2% of GDP expenditures for large scale photovoltaics 2025–2030 3% of GDP expenditures for large scale photovoltaics 2030–2031
Policy 5	3% of GDP expenditures for large scale photovoltaics 2016–2020 2% of GDP expenditures for large scale photovoltaics 2020–2025 1% of GDP expenditures for large scale photovoltaics 2030–2031

## Simulation results

**Fig. 7:**
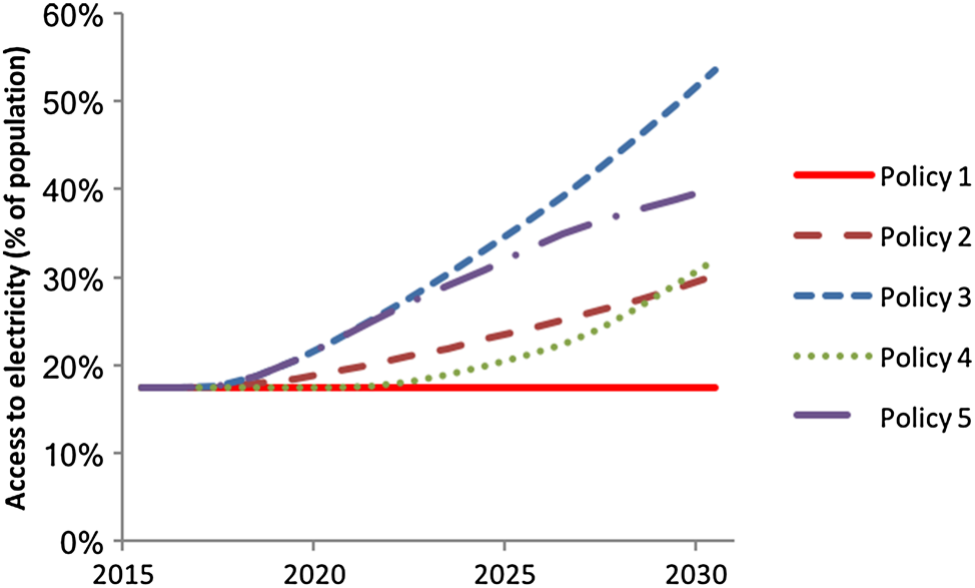
Simulated behaviour of electricity access for the five policy options with the entire iSDG model simulated

**Fig. 8:**
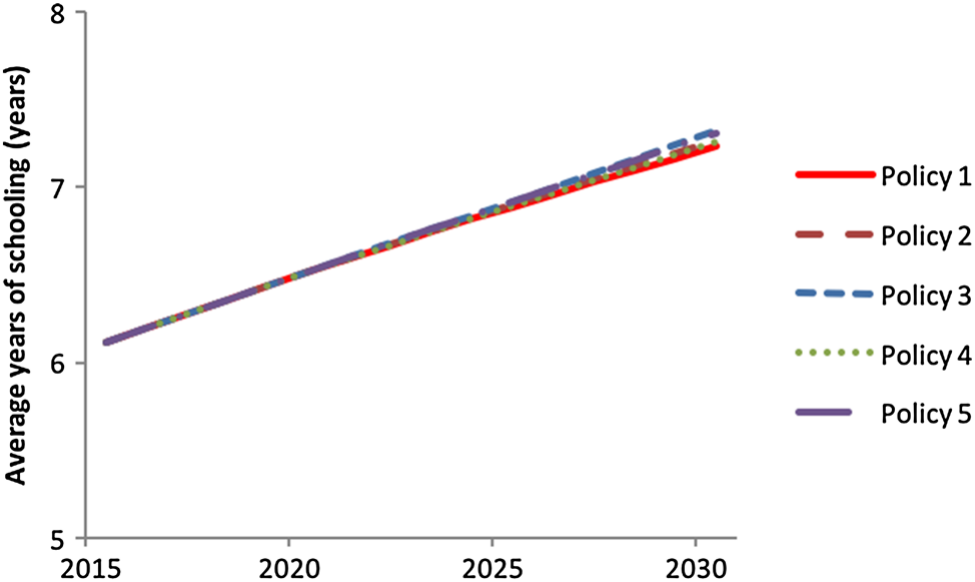
Simulated behaviour of average years of schooling for the five policy options with the entire iSDG model simulated

**Fig. 9:**
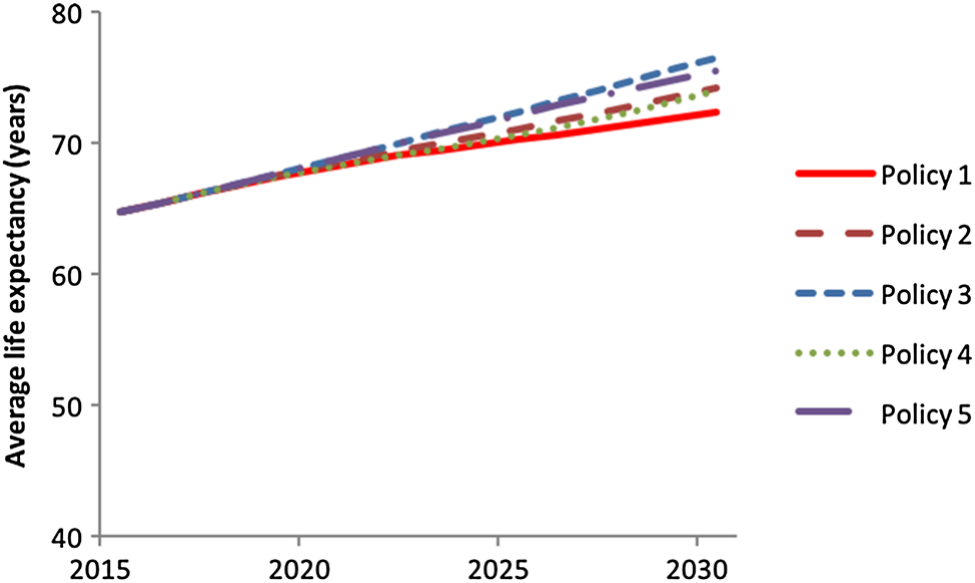
Simulated behaviour of life expectancy for the five policy options with the entire iSDG model simulated

The simulated behaviour of electricity access, years of schooling and life expectancy with the photovoltaics investment policies are presented in Figs. 7, 8 and 9.

The shape of the behaviour of electricity access displayed in Fig. 7 comes as no surprise, as it relates directly to the added investments in photovoltaics in the policy. However, the change is not merely a direct result of these investments. By going back and forth between the model’s causal structure and the simulations, we are able to trace the causalities that affect the model’s overall behaviour. We observe that the effects of the investments are reinforced through their effects on productivity which enables increased future photovoltaic investments (note that investments are added as shares of GDP). Also, the causal chains incorporated in the model that goes via health and education (R1, R2 and R3 in Fig. 6) amplify this reinforcement—the loops synergize. All this contributes to the exponential trend of electricity access for policies 2, 3 and 4.

For average years of schooling (Fig. 8), the simulated differences between the policy options are fairly small. This is because of the long delays incorporated in the model structure related to average years of schooling. Average years of schooling represent the average for the entire population, not just the children currently in school. This means that there is a large adjustment time in response to policy interventions, and changes in average years of schooling play out very slowly. When we consider the effects on lower age cohorts it is greater. Also, there is a saturation effect incorporated in the model’s relationships related to years of schooling, because the number of years of schooling does not continue to rise forever in any country.

With regard to life expectancy, Fig. 9, the differences between the policy scenarios are larger. Life expectancy changes faster than average years of schooling, as the delays in the model structure are shorter. The reasoning behind this is that, while education typically only involves younger age cohorts, a large share of the population is directly affected by health improvements (not least the elderly). A comparison between policies 4 and 5 also indicates that early investments are better than later ones. This is because the earlier investments allow the reinforcing loops to play out for a longer time than investments made later.

## Discussion: the use of integrated planning tools for policy coherence on the SDGs

The research highlights benefits from considering interactions between SDGs in a structured way with the use of integrated simulation tools. Working with the iSDG model brings the multitudes of possible feedback loops that shape a country’s development to the forefront. The model not only maps interlinkages, but also says something plausible about the resulting behaviour of different policy options. The synergies that we have found between SDGs 3, 4 and 7 in Tanzania seem to give rise to system-wide improvements beneficial for goals attainment. The model may also be used to study other causal pathways in which investments in photovoltaics affect development. For example, investments in photovoltaics could also be evaluated with a focus on hypothesized effects on infrastructure. Furthermore, discovering more synergies related to human development might strengthen the case for investments in photovoltaics. However, bottlenecks may also be found, where increased investment does not have the intended effect. The model also allows for studying other policy options and technological investments such as investments to increase agricultural productivity. By comparing plausible results from different interventions, synergies and bottlenecks can be assessed systematically rather than piecemeal.

Our approach illuminates the SDGs interaction framework suggested by Nilsson et al. (2016; also in International Council for Science 2016, and expanded in Nilsson 2017). Using their ratings, our analysis indicates that the improvements in electricity access *enable* progress in educational attainment and life expectancy (+1, “*Creates conditions that further another goal”* 2). Electricity access also causes improvements in life expectancy and years of schooling via productivity increases (higher GDP). We did not find evidence for causal relationships in the opposite direction, from life expectancy and years of schooling to electricity access. These may thereby be rated as *consistent* (0, *“No significant positive or negative interactions”*). Furthermore, the causal relationships between life expectancy and years of schooling are *reinforcing* (+2, *“Aids the achievement of another goal”*), as there is bidirectional causality between the two that does not go via productivity improvements.

The conclusions from the exploration of the iSDG model may also be used in policy planning and to inform public debates. Actual iSDG models may either be used directly in the policy formulation phase, or outsourced to a revision unit that evaluates plausible long-time effects of actual anticipated policies. In both contexts, the model can be used to explore anticipated consequences of different policy options. The iSDG model has been used in a country study on Cote D’Ivoire (Pedercini et al. 2016).

To carefully exploit the many benefits of using integrated models for assessing SDG goals attainment one also has to be cautious of their limitations. There may be unanticipated and unintended consequences of policies that are not included in the scope of the model. Such consequences may affect goal attainment, and the reality will always be more complex than the model and thus incorporate more uncertainties and unforeseen effects. Models can assist us in structuring our thoughts and put light on unintended consequences of different policies, but they do not immunize us against uncertainties and unpredictable real-world behaviours. Also, evidence for many relationships and potential formulations is disputed so alternative model designs always need to be considered. This has been emphasized in an updated version of the Nilsson et al. framework (Nilsson 2017) in which such relationships are discussed.

## Conclusions

We have identified positive causal pathways between educational attainment, life expectancy and electricity access. Integrating these links into the iSDG model initiates reinforcing feedback loops that affect the model’s behaviour. In the simulations, investments in photovoltaics affect both education and health positively, with an enhanced effect caused by synergies in the corresponding feedback structure.

This analysis shows how integrated models can be used to explore systemic relationships between SDGs. It thus demonstrates a flexible, adaptable and suitably transparent approach to generate actionable information that complements the SDG interaction scorings of the Nilsson et al. framework (Nilsson et al. 2016, International Council for Science 2016, and expanded in Nilsson 2017). For models to correspond better to reality and to reflect the ongoing academic and policy debates on integration of SDGs, the behaviour of relevant development indicators needs to be modelled endogenously. Without this, it is difficult to enable broad, cross-sector and long-term analyses of the impact of alternative policies.

Yet integrative modelling is just one part of a shift towards an informed systemic discussion of sustainable development and how best to attain it. An effective analysis process goes beyond the desk study of the published literature and data on causal links to include the exploration of policy options with decision-takers and stakeholders. They bring knowledge of their own contexts that informs the model development and may improve the model’s correspondence to reality.

Research on the attainment of multiple SDGs is growing, but without structured systems understanding there is a risk of repeating the silo approach seen in the implementation of the millennium development goals (Rippin 2014). Integrated tools such as the iSDG model can bring interlinks to the forefront and facilitate a shift to a development discussion based on systems thinking.

## Footnotes

1 Note that the depreciation function averages the lifetime of the constructed photovoltaics. In a more detailed study on the constructed capacity, *photovoltaic capacity* may be separated into different age cohorts based on when the capacity was constructed. The iSDG model allows for such modifications, to reflect technical innovation.

2 International Council for Science (2016) in fact uses the relationship between electricity and education as an example of an enabling relationship.
